# Oculoplastic Conditions in Covid-19 Patients: Case Series and Literature Review

**DOI:** 10.18502/jovr.v19i2.10908

**Published:** 2024-06-21

**Authors:** Camille Yvon, Bhupendra Patel, John Ng, Marcus T Altman, Raman Malhotra

**Affiliations:** ^1^Corneoplastic Unit, Queen Victoria Hospital NHS Trust, East Grinstead, Sussex, United Kingdom; ^2^Moran Eye Center, University of Utah, Salt Lake City, UT, USA; ^3^Casey Eye Institute, Oregon Health and Science University, Portland, OR, USA; ^5^Camille Yvon: https://orcid.org/0000-0003-0959-8777

**Keywords:** Case Studies, Coronavirus, Eyelid Disease, Inflammation, Orbital Disease

## Abstract

**Purpose:**

To investigate oculoplastic conditions in patients diagnosed with coronavirus disease 2019 (COVID-19) seen at ophthalmology departments of three tertiary referral centers in the United Kingdom and the United States, and review of the literature.

**Methods:**

Retrospective multicenter case series studied over 18 months.

**Results:**

A total of four patients developed eyelid, orbital, or lacrimal gland pathology within four weeks of testing positive for COVID-19. All were male, and the mean age at presentation was 49 (range, 31–58 years). Suspected diagnoses included anterior idiopathic orbital inflammation, facial angioedema, dacryoadenitis, and anophthalmic socket inflammation. Three patients recovered fully and one patient recovered partially (mean 2.7 weeks) from ocular manifestations with steroids hastening recovery.

**Conclusion:**

Adnexal manifestations of COVID-19 include self-limiting orbital inflammation and eyelid lymphedema.

##  INTRODUCTION 

The coronavirus disease 2019 (COVID-19) outbreak caused by the severe acute respiratory syndrome coronavirus 2 (SARS-CoV-2) spread throughout the world and was declared a pandemic on March 11 ${\;}^{\text{th}}$ , 2020. It is a highly contagious disease and carries a high case fatality, with global deaths reaching 6.9 million to date.^[1,2,3,4]^

Clinical research regarding COVID-19 has primarily been directed to the respiratory system; a mounting body of evidence describing ocular signs has, however, also emerged.^[5,6,7]^ The prevalence of ocular manifestations in SARS-CoV-2 is estimated to be 11.03%,^[8]^ and a variety of ocular features have been recognized. COVID-19 most commonly affects the anterior segment leading to conjunctivitis, chemosis, and keratitis.^[7,9,10]^ In a cross-sectional study of 535 patients, the most common symptoms were dry eye, blurred vision, and foreign body sensation.^[10]^ Ocular findings may occur early in a minority of patients with COVID-19 infection, even prior to developing systemic symptoms.^[11]^

There have been fewer reports of posterior segment involvement (e.g., vascular occlusions and retinitis), as well as neuro-ophthalmic manifestations (e.g., optic neuritis, cranial nerve palsies).^[5,6,7]^ These findings typically occurred in patients with more severe systemic features. Orbital manifestations in the setting of COVID-19 are uncommon. Numerous studies have reported high rates of COVID-19-associated mucormycosis, particularly affecting patients in the Indian subcontinent.^[12,13,14,15]^ The aim of our study is to present a case series of interesting oculoplastic conditions occurring in the context of COVID-19 infection and review cases published in the medical literature.

##  METHODS

Cases were collected retrospectively based on author recall in tertiary hospitals in the United Kingdom (UK) and United States (US), between March 2020 and September 2021. Inclusion criteria included the diagnosis of an oculoplastic or orbital condition within four weeks of testing positive for COVID-19. Details obtained from the medical record comprised demographics, past medical history, laboratory investigations, radiological findings, and treatment. The study was conducted in compliance with the Declaration of Helsinki and was approved by the local ethics review committee by the registration number of 2030. Consent to publish identifiable photographs was obtained.

##  RESULTS

This clinical case series describes four patients who developed eyelid, orbital, or lacrimal gland pathology following SARS-CoV-2 infection. All patients were unvaccinated at presentation. The clinical and laboratory findings, treatment, and outcomes for the described patients are listed below [Table 1].

### Case 1 

**Figure 1:**
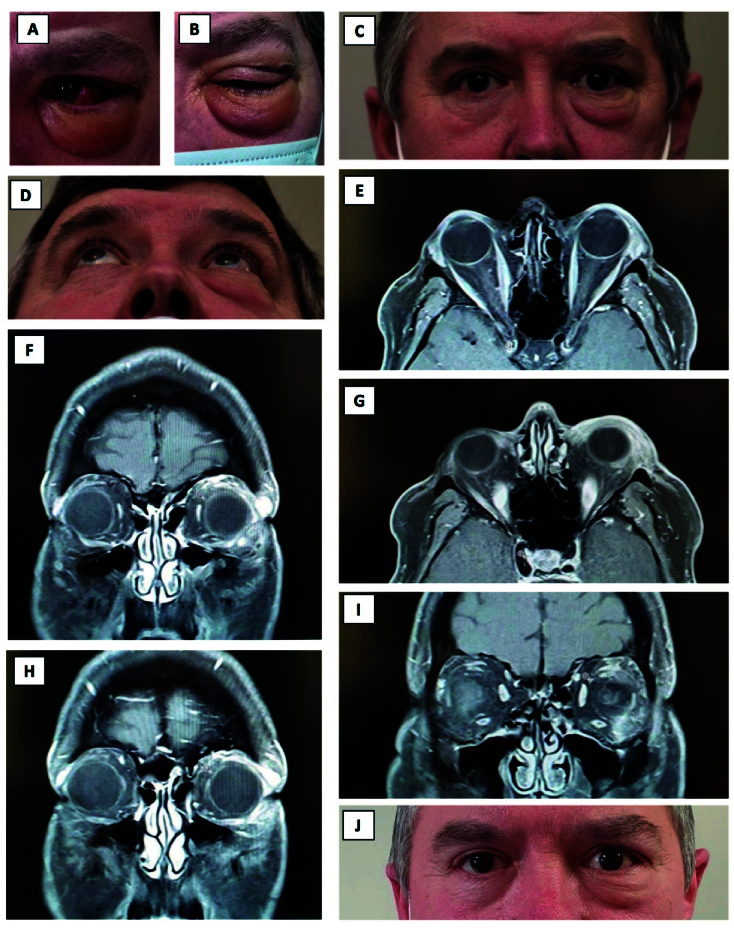
Case 1 (57-year-old male). Photographs showing left lower lid swelling (A–C). T1 weighted MRI head and orbit, axial (E, G) and coronal sections (F, H, I) showing an enlarged lacrimal gland with some ill-defined edges, enhancement, and thickening in the anterior orbit, extending inferotemporal and along the inferior orbital rim, but not involving recti. Photograph taken one-month post intralesional steroid injection (J).

A 57-year-old man developed an acute onset of left lower lid swelling 3.5 weeks after testing positive for COVID-19 while still symptomatic from the infection [Figure 1A-D]. He had no associated pain, itchiness, or redness. Since his COVID-19 illness, the patient had intermittent tiredness, loss of appetite, weight loss following an episode of diarrhea, but no night sweats. He had a background of longstanding mild asthma, controlled with a steroid inhaler in the winter. Examination revealed left lower eyelid lymphedema and a soft non-tender thickening on the left inferior orbital rim, no proptosis, and mild lacrimal gland enlargement.

Magnetic resonance imaging (MRI) showed an enlarged lacrimal gland with ill-defined edges, in addition to enhancement and thickening in the anterior orbit, extending inferotemporal and along the inferior orbital rim, but not involving the recti [Figure 1E-I]. Routine blood tests and thyroid profile were normal. The findings were in keeping with anterior idiopathic orbital inflammation possibly related to his COVID-19 infection.

He underwent a biopsy of his left lacrimal gland and of the soft tissue thickening, which showed mild chronic dacryoadenitis (presence of plasma of cells, immunoglobulin G negative), but no pathology was noted in the specimen from the left orbital rim. As the lower eyelid swelling was persistent, he was treated with three intralesional steroid injections (one month apart) with marginal improvement [Figure 1J].

### Case 2

**Figure 2:**
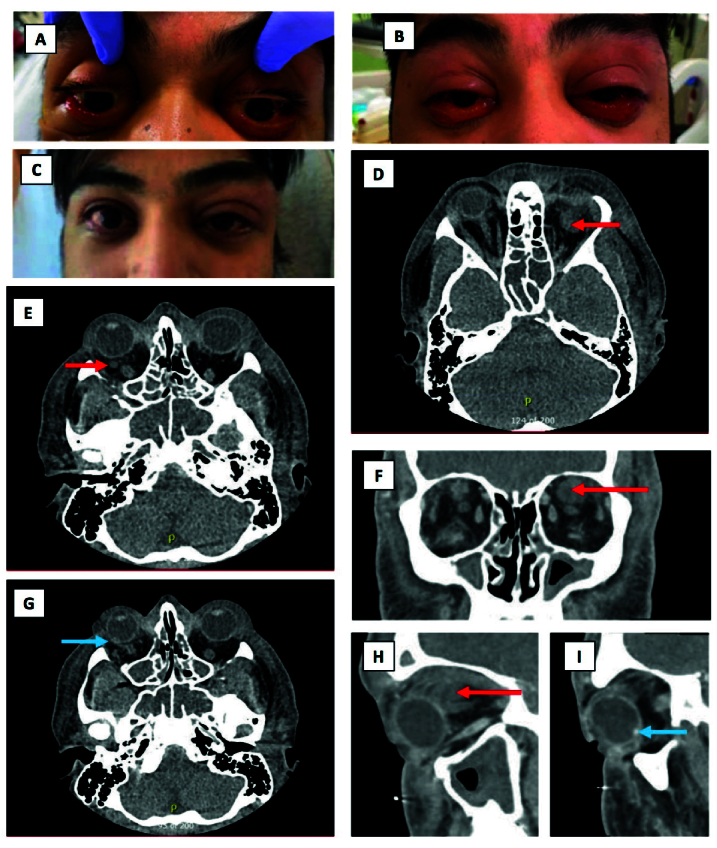
Case 2 (31-year-old male). Photographs showing periorbital edema and conjunctival injection at day 1 (A), day 2 (B), and day 7 of hospitalization (C). CT head and orbit with soft tissue window, axial (D, E, & G), coronal (F), and sagittal (H) sections demonstrating diffuse facial/scalp/periorbital soft tissue thickening and edema with no evidence of orbital abscess. The red arrows show the orbital lesions suspicious for hemorrhage or thrombus, and the blue ones reveal the calcified scleral nodule seen on examination.

A 31-year-old previously healthy man presented with shortness of breath, severe bilateral periorbital, facial and tongue edema, moderate proptosis, and conjunctival chemosis [Figure 2A–2B]. He was ultimately admitted to the medical intensive care unit for acute hypoxemic respiratory failure requiring mechanical ventilation and multifactorial shock in the setting of SARS-CoV-2 pneumonia. He also had Streptococcus anginosus bacteremia, acute systolic heart failure from sepsis-induced cardiomyopathy, immune thrombocytopenia (ITP), with a platelet count of 7 on admission. He later developed culture-positive multifocal brain abscesses from Porphyromonas endodontalis.

Computerized tomography (CT) of orbits showed diffuse facial/scalp/periorbital soft tissue thickening and edema. There were no discreet masses or evidence of orbital cellulitis, abscess, or cavernous venous thrombosis. No other etiology could be determined for the cause of the edema. He was ultimately diagnosed with a clinical syndrome of possible COVID-related facial angioedema.

He did, however, have two orbital lesions suspicious for localized orbital hemorrhage or thrombus in the setting of his ITP [Figure 2D–2I]. Fundus examination also revealed an elevated peripheral retinal lesion in the right eye, which could be seen radiographically on CT as a hyperdense posterior globe lesion [Figure 2G & 2I]. This finding was thought to be a choroidal/scleral nodule or thrombosed vortex vein and resolved rapidly on a subsequent follow-up exam and B-scan several days later. Further imaging including angiography was therefore not pursued.

The severity of the edema required treatment with epinephrine for possible anaphylaxis. The patient also received COVID-19 treatments, including intravenous (IV) dexamethasone and medications as part of a COVID-19 clinical trial (infliximab/placebo + remdesivir × 5 days). His periorbital/ocular edema completely resolved over the subsequent one to two weeks [Figure 2C].

### Case 3

**Figure 3:**
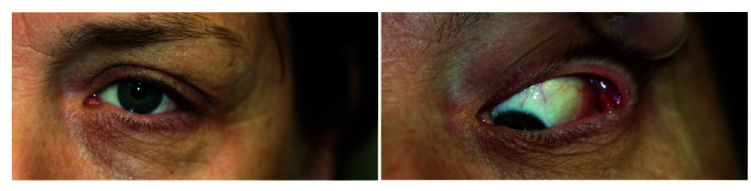
Case 3 (54-year-old male). Photographs showing left S-shaped upper lid swelling.

A 54-year-old man presented with a four-week history of left S-shaped upper lid swelling with moderate tenderness, which started while being treated as an inpatient with COVID-19 pneumonia [Figure 3]. He was discharged from the hospital without an ocular examination but presented to our ophthalmic department as the area had become more tender. His vision remained stable, and he denied any diplopia. Examination showed tenderness over a palpable lacrimal gland, no proptosis, and mild conjunctival chemosis. He was treated with systemic broad-spectrum antibiotics, and the swelling resolved within two weeks.

### Case 4

**Figure 4:**
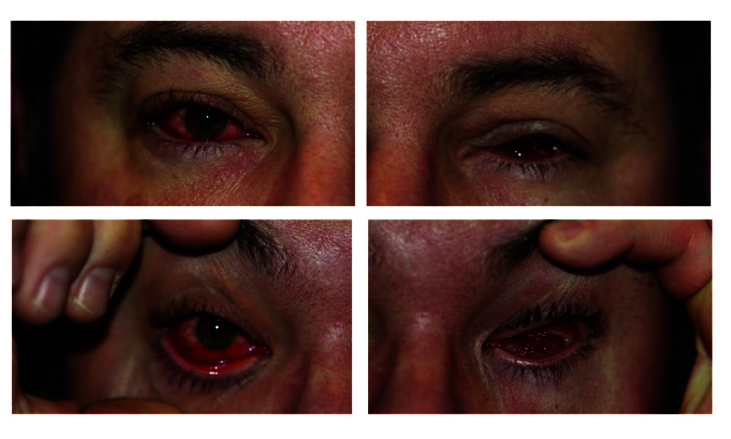
Case 4 (43-year-old male). Photographs showing a right injected eye and left inflamed anophthalmic socket with some discharge.

A 43-year-old man who had a previous left enucleation 12 years ago due to a melanoma presented with a right red, watery eye, in addition to a left inflamed socket with difficulty wearing the prosthesis for two weeks and pain on movement of the left socket contents [Figure 4]. He tested positive for SARS-CoV-2 three weeks previous to the commencement of symptoms. Examination via an online platform showed socket injection and inflammation, with a marked papillary reaction. He was treated with topical broad-spectrum antibiotics (ciprofloxacin), and the inflammation resolved in three weeks.

##  DISCUSSION 

Our case series highlights four uncommon orbital presentations occurring in the context of SARS-CoV-2 infection. These four cases, presenting in different countries, share similar clinical features, and all were SARS CoV-2 RNA positive. It is difficult to confirm whether SARS-CoV-2 was coincidental or a contributing factor to the pathogenesis. Nevertheless, the temporal association and exclusion of other causes point toward orbital inflammatory changes secondary to a COVID-19 infection.

Orbital and adnexal manifestations of COVID-19 are unusual; however, studies continue to emerge on further links [Table 2]. In a meta-analysis reviewing 1021 COVID-19 positive patients with ocular signs, 8 cases (0.9%) of eyelid edema were reported.^[8]^ There have been several reports of facial swelling associated with SARS-CoV-2, which typically responded to steroids and/or antihistamines, depending on severity.^[16,17,18,19]^ Armstrong et al^[20]^ described a case of suspected orbital myositis associated with COVID-19. The patient did not respond to systemic antibiotics but improved significantly following IV and oral corticosteroids. Studies have demonstrated that ocular signs are seen more commonly in patients with higher white blood cell count, C-reactive protein, procalcitonin, and lactate dehydrogenase levels.^[7,21]^

**Table 1:** Summary table of clinical findings and treatment.


Patient	1	2	3	4
Location	UK	US	US	US
Age	57	31	54	43
Gender	Male	Male	Male	Male
Clinical features	4/52 Hx of non-tender thickening of orbital rim; enlarged lacrimal gland on MRI	1/52 Hx of bilateral periorbital and facial edema; moderate proptosis; conjunctival chemosis	4/52 Hx of S-shaped swelling of upper eyelid; tender lacrimal gland	2/52 Hx of right watery, injected eye and left inflamed socket and unable to fit prosthesis
Diagnosis	Anterior idiopathic orbital inflammation/mild chronic dacryoadenitis	Facial angioedema	Dacryoadenitis	Inflammation of anophthalmic socket
Other symptoms	Intermittent tiredness, weight loss	Pneumonia; hypoxemic respiratory failure requiring mechanical ventilation; multifactorial shock; ITP, multifocal brain abscess	Recent hospitalization for COVID-19	N
Treatment				
Antibiotic	N	N	Y	Ciprofloxacin eye drops (improved)
Antiviral	N	Infliximab / placebo + remdesivir x5days	N	N
Steroid	3 × intralesional steroid injections to rim (thickening partially improved)	IV dexamethasone (improved)	N	N
	
	
Hx, history; ITP, immune thrombocytopenic purpura; IV, MRI, magnetic resonance imaging; intravenous; N, no/none; UK, United Kingdom; US, United States				

**Table 2:** Literature review of oculoplastic and orbital manifestations of COVID-19


**Study**	**Type**	**Location**	**Sample**	**Age (mean)**	**Diagnosis**	<@orange**Treatment response**		
			**Antibiotics/ Antiviral antifungals**	**Corticosteroids**	**Other**
Armstrong et al^[20]^	Case report	USA	1	44	Orbital myositis	Systemic antibiotics - *poor response*	Intravenous followed by oral steroids - *very good response*	
Martínez Diaz et al^[22]^	Case report	Spain	1	22	Acute dacryoadenitis + partial ophthalmoplegia	Oral amoxicillin-clavulanic acid – *poor response*	Oral high-dose prednisone *– very good response*	Dexketoprofen – *poor response*
Turbin et al^[23]^	Case report	USA	2	12	Orbital cellulitis	Parenteral vancomycin, ceftriaxone, metronidazole, topical ocular tobramycin ointment – *good response*	Fluticasone nasal sprays	
		15	Orbital cellulitis + intracranial epidural abscess and thrombophlebitis of superior ophthalmic vein	Parenteral vancomycin, ceftriaxone, metronidazole – *moderate response *	Endoscopic frontal sinusotomy, total ethmoidectomy and maxillary antrostomy + levetiracetam for seizure prophylaxis
Shires et al^[24]^	Case report	USA	1	76	Orbital abscess + osteomyelitis (*Peptoniphilus indolicus)*	Intravenous antibiotics – *short-lived*	Endoscopic transnasal drainage and orbitotomy Enucleation 2/12 later
Sen et al^[37]^	Retrospective, observational study	India	2826	52	Orbital mucormycosis	Intravenous (73%) +/– intraorbital injection (22%) of amphotericin B *14% mortality *	Functional endoscopic sinus surgery/paranasal sinus debridement 56%, exenteration 15%, combination 17% *Paranasal sinus debridement and orbital exenteration reduced the mortality rate from 52% to 39%*
Singh et al^[12]^	Retrospective, observational study	India	187	57	Orbital mucormycosis	Antifungals (liposomal amphotericin B alone or combination therapy) *46% mortality at 12 weeks*	Major resection (21%); partial resection or debridement (79%)
Selarka et al^[28]^	Prospective, observational study	India	47	55	Orbital mucormycosis	100% empirical broad ‐ spectrum antibiotics + anti ‐ viral medication (Remdesivir or Favipiravir) + antifingals *11/47: non-survivors*	29/47: intravenous steroids 45/47: oral steroids	3/47: immunomodulatory agents, e.g., Tofacinib, intravenous immunoglobulin, and Bevacizumab
Mishra et al^[13]^	Prospective, observational study	India	32	58	Orbital mucormycosis	Amphotercin B *4/32: non-survivors 23/32: still inpatients on parenteral antifungals*	30/32: Endoscopic debridement
	
	
Note: List is not exhaustive for COVID*-*19*-*associated mucormycosis.								

The lacrimal gland may also be affected, leading to pain in the superolateral orbit and the characteristic S-shaped curve of the eyelid margin. Martínez Diaz et al^[22]^ described a case of acute dacryoadenitis in a 22-year-old male with positive SARS-CoV-2 antibodies who developed partial ophthalmoplegia. The patient was otherwise devoid of COVID-19 symptoms. He was initially treated with oral amoxicillin-clavulanic acid and dexketoprofen, but the periocular inflammation increased. He was treated with oral prednisone (60 mg daily) and rapidly improved. The dose was subsequently tapered down over a month.

Turbin et al^[23]^ described two cases of orbital cellulitis in adolescent patients who tested positive for COVID-19. Both patients improved with parenteral vancomycin, ceftriaxone, metronidazole, topical ocular tobramycin ointment, fluticasone, and oxymetazoline nasal sprays. The second patient also received enoxaparin, hydroxychloroquine, and levetiracetam, as he was found to have an intracranial epidural abscess and thrombophlebitis of the right superior ophthalmic vein.

Unusual pathological findings have been reported in COVID-19 positive patients. Shires et al^[24]^ described a case of orbital abscess caused by *Peptoniphilus indolicus*, normally a commensal of the vagina or gut. He was treated with IV antibiotics and discharged but then presented two months later with worsening symptoms. Examination revealed multiple scleral perforations as well as orbital abscesses. He subsequently required an enucleation and excision of the necrotic orbital bone.

There have been numerous case reports of invasive mucormycosis in the context of COVID-19^[25,26,27]^ and large case series mainly originating from the Indian subcontinent.^[12,13,14,15]^ The incidence and prevalence of COVID-19-associated mucormycosis have risen rapidly during the second wave, with the state of Gujarat in India reporting the highest number.^[28,29,30]^ Mucormycosis affects immunocompromised patients, particularly those with diabetes mellitus, prolonged corticosteroid use, solid organ transplant recipients, and hematological malignancies. In a COVID-19 setting, the main risk factors comprise hyperglycemia, previous mechanical ventilation, and non-receipt of the COVID-19 vaccine.^[28,31]^ Systemic corticosteroids are often included in the treatment of SARS-CoV-2. Its use in poorly controlled diabetics may also contribute to the high rates of mucormycosis.^[13,32]^

The prevalence of mucormycosis is significantly higher in India compared to the developed world.^[33]^ This is likely due to the higher numbers of thermotolerant saprophytic fungi (known as Mucorales*)* typically found in soil or decaying vegetation. Mucorales has also been detected in air samples in outdoor and indoor settings (including hospitals) in north India,^[34]^ and rarer species such as *Apophysomyces *have been isolated in India's nitrogen low soil.^[35]^ Other contributing factors include the large number of immunocompromised hosts, particularly undiagnosed diabetics, the neglect for regular health check-ups, and the lower COVID-19 vaccination rate.^[36]^

Sen et al^[37]^ analyzed the data of 2826 patients with COVID-19-associated mucormycosis. Clinical features at presentation comprised headache, facial edema, ptosis, ophthalmoplegia, proptosis, and loss of vision.^[37]^ The most commonly involved sinus on radiological imaging was the maxilla, followed by the ethmoid, sphenoid, and frontal. Despite treatment with antifungals and surgery, COVID-19-associated mucormycosis carries a low prognosis, with an average mortality rate of 31%.^[37,38]^ This is mainly due to mycotic thrombosis, ischemic infarction, and tissue necrosis.^[13,28]^

Numerous mechanisms have been proposed to be the cause of these orbital inflammatory changes. The development of eyelid edema may be associated with angiotensin-converting enzyme 2 (ACE-2), which plays a crucial role in inhibiting the potent endothelium-dependent vasodilator bradykinin. Studies have shown that SARS-CoV-2 enters the host cells by binding to ACE-2 and subsequently impedes the breakdown of bradykinin and its mediators.^[39]^ This is thought to cause overstimulation of the bradykinin pathway, resulting in increased vascular leakage and angioedema.^[40]^ These postulated mechanisms are comparable to those in acute respiratory distress syndrome, where the virus triggers acute pulmonary edema.

The orbital inflammatory changes described in our case series are likely manifestations of viral infection. SARS-CoV-2 may act like other viruses by inducing a state of mast cell activation, resulting in interleukin (IL) and histamine release.^[41]^ This is thought to be a cross-reaction between viral IgM and IgG, with mast cell IgE favoring mast cell degranulation. Furthermore, cytokines that are often released in COVID-19, including IL-6, IL-1 β , and interferon-gamma, are also potent mediators of inflammation and may predispose to the development of idiopathic orbital inflammation.^[42]^ The virulence is possibly caused by a cytokine storm and vascular permeability.^[43]^

The pathophysiology of dacryoadenitis secondary to COVID-19 may differ slightly. It is thought that SARS-CoV-2 spreads via the lacrimal ductules or hematogenously and subsequently induces an immune-inflammatory response.^[22]^ Interestingly, ACE-2 has been identified in extrapulmonary tissues, including lacrimal glands in murine models.^[44,45]^ Early evidence has identified SARS-CoV-2 in ocular secretions,^[46,47]^ and it is thought to spread to the respiratory tract via the tear drainage pathway. Deng et al^[48]^ showed that SARS-CoV-2 was detected early in the conjunctiva swab of inoculated rhesus macaques, who then developed mild interstitial pneumonia.

There are no current guidelines for the management of ocular inflammatory changes in SARS-CoV-2-positive patients. It is difficult to know whether the treatment had a direct effect or whether the resolution was consistent with the natural course of untreated eyelid edema. Steroids seemed to hasten recovery in severe cases; however, it is critical to use the drug cautiously due to its prothrombotic properties. The effectiveness and safety of corticosteroids remain uncertain.^[49,50]^ Recently, the Randomized Evaluation of COVID-19 Therapy (RECOVERY) collaborative revealed lower mortality rates when adding a low daily dose of dexamethasone (for up to 10 days) to the treatment regime of SARS-CoV-2-positive patients on oxygen or mechanical ventilation.^[51]^ The positive effects of dexamethasone may have been attenuated by the use of an effective antiviral agent and subsequent viral control.

Post-COVID syndrome^[52]^ and long COVID are terms used to describe the continuation of symptoms in those who have recovered from SARS-CoV-2 infection. The condition typically occurs beyond 12 weeks after infection.^[53]^ The majority of patients are PCR negative, in keeping with microbiological recovery. Most common symptoms involve fatigue, myalgia, weight loss, dyspnea, cough, chest pain, and memory deficit. It has been hypothesized that a pro-coagulant state, cytokine storm, cellular damage,^[54]^ and small fiber neuropathy may underlie long COVID.^[55]^ Bitirgen et al^[56]^ found corneal nerve fiber loss in patients with this syndrome. To date, we have no evidence that long COVID increases the likelihood of later orbital involvement.

Our study has a few limitations, including its small size, recall bias, and one patient still undergoing active treatment. Furthermore, it is challenging to confirm whether SARS-CoV-2 was coincidental or a contributing factor to the disease. We acknowledge that this is a hypothetical relationship between COVID-19 and the aforementioned conditions. Additional studies are warranted to examine the underlying mechanism of ophthalmic complications of SARS-CoV-2. Corticosteroids are part of the armamentarium against COVID-19 but must be used carefully. Future studies should address the optimum therapy for oculoplastic conditions in the setting of COVID-19.

In conclusion, SARS-CoV-2 is efficiently transmitted and presents broad tissue tropism, which likely contributed to the pandemic and emergence of new variants. Our study draws attention to four uncommon orbital presentations occurring in the context of COVID-19 coinfection. It is essential to rule out other underlying causes to establish the diagnosis. Most ocular complications of COVID-19 are likely inflammatory in origin. Corticosteroids can be considered in the treatment after excluding infectious causes such as Mucor. Early recognition of these clinical findings would facilitate diagnosis, track and trace and subsequently reduce transmission.

##  Declaration of Patients Consent

The authors certify that they have obtained all appropriate patients consent forms. In the form the patients have given their consent for thier images and other clinical information to be reported in the journal. The patients understand that thier names and initials will not be published and due efforts will be made to conceal their identities, but anonymity cannot be guaranteed.

##  Financial Support and Sponsorship

The study is supported in part by an unrestricted grant from Research to Prevent Blindness, Inc., New York, NY, to the Department of Ophthalmology & Visual Sciences, University of Utah and grant from Research to Prevent Blindness, Inc., New York, NY, to Casey Eye Institute, Oregon Health and Science University.

##  Conflicts of Interest

None.
